# Effects of Different G-Protein α-Subunits on Growth, Development and Secondary Metabolism of *Monascus ruber* M7

**DOI:** 10.3389/fmicb.2019.01555

**Published:** 2019-07-09

**Authors:** Ming Lei, Jiao Liu, Yang Fang, Yanchun Shao, Li Li, Jae-Hyuk Yu, Fusheng Chen

**Affiliations:** ^1^Hubei International Scientific and Technological Cooperation Base of Traditional Fermented Foods, College of Food Science and Technology, Huazhong Agricultural University, Wuhan, China; ^2^Institute of Quality Standard and Testing Technology for Agro-Products, Hubei Academy of Agricultural Sciences, Wuhan, China; ^3^National Engineering Research Center for Natural Medicines, Chengdu, China; ^4^College of Life Science, Yangtze University, Jingzhou, China; ^5^Departments of Bacteriology and Genetics, University of Wisconsin – Madison, Madison, WI, United States; ^6^Department of Systems Biotechnology, Konkuk University, Seoul, South Korea

**Keywords:** *Monascus ruber*, G-protein α-subunit, development, secondary metabolism, transcriptomic analysis

## Abstract

Strains of *Monascus* filamentous fungal species have been used to produce fermented foods in Asian countries, such as China, Japan, and The Korean Peninsula, for nearly 2,000 years. At present, their fermented products are widely used as food additives and nutraceutical supplements worldwide owing to their production of beneficial secondary metabolites. Heterotrimeric G-protein signaling pathways participate in regulating multiple biological processes in fungi. Previously, we identified three *Monascus ruber* M7 G-protein α subunits (Mga1–3) and demonstrated that Mga1 can regulate growth, reproduction and some secondary metabolites’ production. Here, we systematically analyzed and compared the roles of *mga*1–3 by combining single- and double-gene(s) knockouts and their transcriptomic data. First, *mga*2 and *mga*3 knock-out mutants and pairwise combinations of *mga*1–3 deletion strains were generated. Then the changes in growth, development and the main secondary metabolites, *Monascus* pigments and citrinin, in these mutants were systematically compared with *M. ruber* M7. Moreover, RNA-Seq analyses of these mutants were performed. All three Gα subunits worked together to regulate biological processes in *M. ruber* M7, with Mga1 playing a major role, while Mga2 and Mga3 playing supplemental roles. According to the existing literatures which we can find, gene knock-out mutants of the pairwise combination of *mga*1–3 and their transcriptome analysis are first reported in this study. The current results have clearly demonstrated the functional division of Mga1–3 in *M. ruber* M7, and could provide a deeper understanding of the effects of different Gα subunits on growth, development and secondary metabolism in other filamentous fungi.

## Introduction

*Monascus* spp. have been used in food fermentation in Asian countries, such as China, Japan, and The Korean Peninsula, for nearly 2,000 years (Chen et al., 2015; Kim et al., 2016; Rahayu et al., 2017). At present, their fermented products, such as red fermented rice (RFR), also called red yeast rice, *Anka*, *Hongqu*, red koji, and red mold rice, are widely used as food coloring, fermentation starters and food supplements worldwide (Chen et al., 2015; Yu et al., 2015; Wei et al., 2017; Derosa et al., 2018; Luo et al., 2018), because *Monascus* spp. can produce various useful secondary metabolites (SMs), mainly including *Monascus* pigments (MPs), monacolin K (MK) and γ-amino butyric acid (Feng et al., 2012; Huang et al., 2013; Patakova, 2013; Diana et al., 2014). However, some strains of *Monascus* spp. may also produce citrinin (CIT), a kind of nephrotoxic mycotoxin (Blanc et al., 1995). Therefore, how to increase the beneficial SMs levels and reduce the CIT level in RFR has become the research hotspot over the last 10 years (Jia et al., 2010; Li et al., 2013; Liu et al., 2014; He and Cox, 2016; Alberti et al., 2017).

The genes involved in SMs biosynthesis in filamentous fungi usually appear as gene clusters (Schwecke et al., 1995; Blin et al., 2015). In the past decade, the gene clusters of MPs, CIT and MK in *Monascus* spp. have been identified and their biosynthetic pathways have been fully illustrated (Chen et al., 2008; Li et al., 2015; He and Cox, 2016; Liu J. et al., 2016; Chen et al., 2017). The biosynthesis of these SMs cannot only be controlled by the intra-cluster regulating genes but can also be adjusted by the off-cluster global regulating genes, such as *Lae*A, *Ve*A, and related genes in the G-protein signaling pathway (GPSP) (Fox and Howlett, 2008; Liu Q. et al., 2016; Lin et al., 2018). GPSPs, including the G-protein coupled receptor (GPCR), heterotrimeric G-protein (G-protein) and downstream effectors (Seo and Yu, 2006), play vital roles in growth, differentiation, SMs biosynthesis, pathogenicity and toxicity in filamentous fungi (Yu et al., 2008; Corrochano et al., 2016; Moretti et al., 2017; Liu et al., 2018; van den Hoogen et al., 2018).

Each G-protein generally composes α, β and γ subunits. In most characterized filamentous fungi, the Gα proteins are classified as three groups (Gα1–3) (Li et al., 2007). Although the functions of individual Gα subunits have been well investigated in model and pathogenic fungi (Li et al., 2010; Yang et al., 2012; Hu et al., 2013; Wasil et al., 2013; Garcia-Rico et al., 2017), there is limited research on the interplay among different Gα subunits (Kamerewerd et al., 2008).

In our previous study, the roles of the G protein α subunit gene *mga*1 (Gα 1 gene) in wild-type *M. ruber* M7 were analyzed, and *mga*1 can comprehensively regulate growth, reproduction, and MPs and CIT production (Li et al., 2010). Here, the other two Gα genes, *mga*2 (Gα 2 gene) and *mga*3 (Gα 3 gene), and the pairwise combinations of *mga*1–3 were independently deleted in *M. ruber* M7. The morphological observations and fermentation experiments of six Gα genes’ mutants, Δ*mga*1, Δ*mga*2, Δ*mga*3, Δ*mga*1+2, Δ*mga*1+3, and Δ*mga*2+3, as well as their RNA-Seq analyses, were conducted to systematically investigate the functions of the Gα subunits in *M. ruber* M7. And we have found that all three Gα subunits work together to regulate extensive biological processes in *M. ruber* M7, Mga1 playing a major role and Mga2 and Mga3 as supplementary roles. In detail, during vegetative growth, Mga1 is the essential positive regulator, while Mga2 and Mga3 can enhance the regulatory process when either was double deleted with Mga1. However, a single deletion of Mga2 or Mga3 has little effect. Mga1 contributes the most to the regulation of sexual/asexual reproduction, and the regulation of asexual reproduction may occur prior to the central regulatory pathway. Different Gα subunits can be combined to negatively regulate secondary metabolism. Mga1 and Mga2 can negatively regulate MPs and CIT production individually or jointly, and Mga3 may work in combination with Mga1 to negatively enhance regulation of MPs production. These findings not only illuminate the functions of different Gα subunits in *M. ruber* M7 but could also provide a deeper understanding of the effects of different Gα subunits on growth, development and secondary metabolism in other filamentous fungi.

## Materials and Methods

### Strains and Media

*Monascus ruber* M7 (CCAM 070120, Culture Collection of State Key Laboratory of Agricultural Microbiology, China Center for Type Culture Collection, Wuhan, China) (Chen and Hu, 2005) was used to generate the gene knockout strains Δ*mga*2 and Δ*mga*3. The Δ*mga*1 strain obtained by Li in our laboratory (Li et al., 2010) was used to generate the double-deletion strains Δ*mga*1+2 and Δ*mga*1+3. The Δ*mga*2 strain obtained in this study was used to generate the double-deletion strain Δ*mga*2+3.

Potato dextrose agar (PDA), malt extract agar (MA), czapek yeast extract agar (CYA) and 25% glycerol nitrate agar (G25N) were utilized for phenotypic characterization (He et al., 2013). PDA was used for the analyses of MPs and CIT production. G418 (Sigma-Aldrich, Shanghai, China) or hygromycin (Sigma-Aldrich, Shanghai, China) was added to the medium for transformant selection (Yang et al., 2012).

### Deletion of Gα Genes in *M. ruber* M7

The homologous gene recombination strategy was used to construct the deletion strains (Δ*mga*2, Δ*mga*3, Δ*mga*1+2, Δ*mga*1+3, and Δ*mga*2+3). The hygromycin resistence gene *hph* was used in the *mga*2-deletion cassette to construct the Δ*mga*2 strain, while the G418 resistence gene *neo* was used in the *mga*3-deletion cassette to construct the Δ*mga*3 strain. The Δ*mga*1 strain had been constructed previously (Li et al., 2010). The Δ*mga*1 strain and another *mga*2-deletion cassette with the *neo* gene were used to construct the double-deletion strain Δ*mga*1+2. The Δ*mga*1 strain and the *mga*3-deletion cassette were used to construct the double-deletion strain Δ*mga*1+3. The Δ*mga*2 strain and the *mga*3-deletion cassette were used to construct the double-deletion strain Δ*mga*2+3. The gene deletion cassette was constructed by double-joint PCR, as shown in Supplementary Figures S1, S2, using the primers listed in Supplementary Table S1. The construction strategy for the complementary strains is also shown in Supplementary Figure S1. The mutants were generated using an *Agrobacterium tumefaciens*-mediated transformation method that was previously established in our laboratory (Li et al., 2010). The genotypes of deletion strains were confirmed using PCR amplification and Southern hybridization.

### Southern Hybridization

Southern hybridization was performed according to a previously reported method (Liu et al., 2014) using a DIG-High Prime DNA Labeling and Detection Starter Kit I (Roche, Germany). Fragments of *mga2* [open reading frame (ORF), probe 1], *hph* (selective marker gene, probe 2), *mga*3 (ORF, probe 3), and *neo* (selective marker gene, probe 4) were independently amplified to be used as probes. The single-deletion mutants’ DNAs were digested by *Sac*I and *Xho*I. The double-deletion mutants’ DNAs were digested by *Kpn*I. Primers are listed in Supplementary Table S1.

### Phenotypic Analysis

*Monascus ruber* M7, Δ*mga*1, Δ*mga*2, Δ*mga*3, Δ*mga*1+2, Δ*mga*1+3, and Δ*mga*2+3 strains were cultivated on PDA, CYA, MA and G25N at 28°C to observe their phenotypes. The colony sizes of these strains were measured after cultivated for 12 days, and the cleistothecia or conidia were observed and counted after cultivated for 5 days. Three replicates are for each strain.

Freshly harvested conidiospores (10^5^ conidia mL^–1^) of *M. ruber* M7 and Gα-deleted strains were inoculated on PDA medium, covered with cellophane and incubated at 28°C for 11 days. The mycelia and medium were sampled every other day from 3 to 11 days to analyze the intracellular and extracellular MPs and CIT production levels (Li et al., 2014).

*Monascus* pigments were determined by their UV-Vis spectra (Agilent Cary 60, Australia). CIT was determined by Waters ACQUITY UPLC I-class system (Waters, Milford, MA, United States) with an ACQUITY UPLC BEH C18 column (2.1 mm × 100 mm, 1.7 μm) according to the established method in our laboratory (Liu J. et al., 2016).

### RNA-Seq Analysis

*Monascus ruber* M7 and Gα mutant strains were independently inoculated on PDA medium, covered with cellophane and incubated at 28°C. Two replicates were conducted for each strain. At 3 and 7 days, mycelia were collected for RNA extraction and then sequenced using the BGIseq-500RS platform (BGI, Wuhan, China^1^). The expression levels of 11 randomly selected genes in *M. ruber* M7 and Δ*mga*1+3 were assessed using quantitative real-time PCR (qRT-PCR) to confirm the reliability of the RNA-Seq results.

*Monascus ruber* M7 genome which contains 8,407 genes, was used as a reference genome (Chen et al., 2015) to calculate the BLAST rate of the genome, and clean data were aligned using Hierarchical Indexing for Spliced Alignment of Transcripts and bowtie2 (Langmead and Salzberg, 2012; Kim et al., 2015). Then, RNA-Seq by Expectation Maximization was used to calculate the expression level of each gene (Li and Dewey, 2011). The genes that possessed an expression differential multiple greater than 1, as well as a *Q*-value not greater than 0.001 (Benjamini and Hochberg, 1995; Storey and Tibshirani, 2003), were selected as differentially expressed genes (DEGs).

Gene ontology (GO)^2^ and a KEGG pathway^3^ functional analysis were used to investigate the functions of the DEGs between *M. ruber* M7 and Gα mutants. Moreover, the DEGs involved in fungal growth, sporulation and secondary metabolism were further analyzed to determine the roles of Gα subunits in development and secondary metabolism of *M. ruber* M7.

## Results

### Targeted Deletion of Gα Genes in *M. ruber* M7

Single-deletion strains Δ*mga*2 with hygromycin resistance and Δ*mga*3 with G418 resistance, as well as double-deletion strains Δ*mga*1+2, Δ*mga*1+3, and Δ*mga*2+3, were obtained. For Δ*mga*2 strain, the PCR analysis confirmed the existence of the *hph* sequence as well as the absence of the *mga*2 ORF. Southern hybridization showed a single copy of the *hph* sequence in the Δ*mga*2 strain. For Δ*mga*3 strain, the PCR analysis confirmed the existence of the *neo* sequence as well as the absence of the *mga*3 ORF. Southern hybridization showed a single copy of the *neo* sequence in the Δ*mga*3 strain. For Δ*mga*1+2 strain, the PCR analysis confirmed the existence of the *hph* and *neo* sequence as well as the absence of the *mga*1 and *mga*2 ORF. Southern hybridization showed a single copy of the *neo* sequence in the Δ*mga*1+2 strain. For Δ*mga*1+3 strain, the PCR analysis confirmed the existence of the *hph* and *neo* sequence as well as the absence of the *mga*1 and *mga*3 ORF. Southern hybridization showed a single copy of the *neo* sequence in the Δ*mga*1+3 strain. For Δ*mga*2+3 strain, the PCR analysis confirmed the existence of the *hph* and *neo* sequence as well as the absence of the *mga*2 and *mga*3 ORF. Southern hybridization showed a single copy of the *neo* sequence in the Δ*mga*2+3 strain. The results of PCR analysis and Southern hybridization were displayed in Supplementary Figures S1, S2. Additionally, the corresponding complementation strains were also obtained. The complementation strains possessed phenotypic characteristics similar to those of *M. ruber* M7 (Supplementary Figure S3).

### Phenotypic Characterization of *M. ruber* M7 and Gα Mutants

#### Vegetative Growth and Reproduction

**FIGURE 1:**
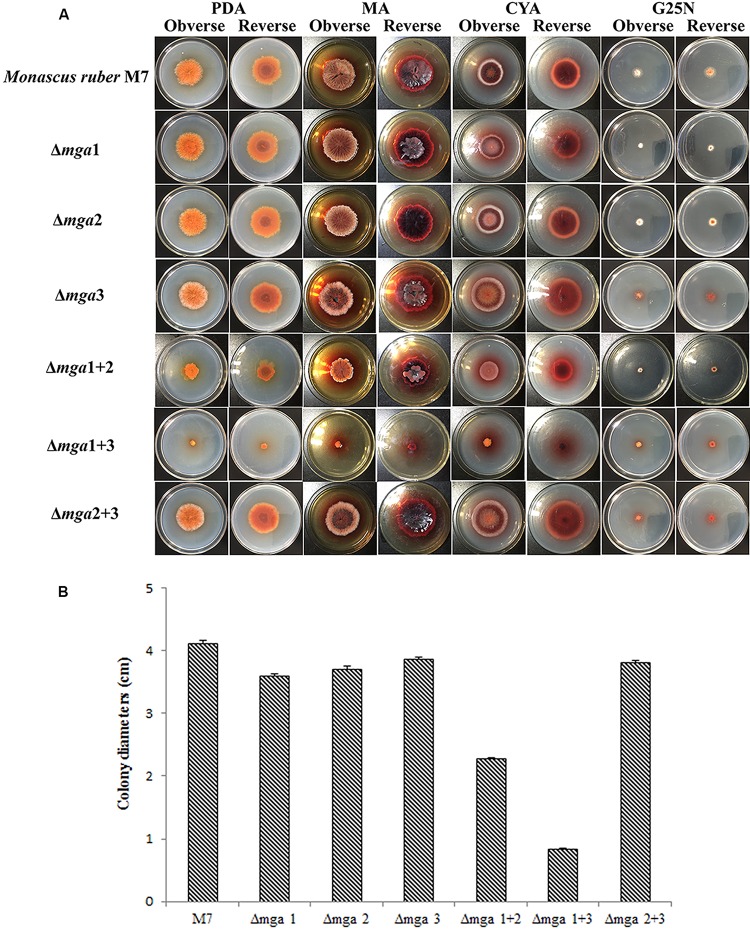
Colony morphologies of Gα mutants and *Monascus ruber* M7. **(A)** Colony morphologies of M7 and Gα mutants observed on PDA, MA, CYA, and G25N plates and cultured at 28°C for 12 days. **(B)** Colony sizes of the indicated strains on PDA medium cultured at 28°C for 12 days.

The phenotypes of the six Gα mutants, Δ*mga*1 (prepared by Li et al. (2010)), Δ*mga*2, Δ*mga*3, Δ*mga*1+2, Δ*mga*1+3, and Δ*mga*2+3, were compared with *M. ruber* M7. As shown in Figure 1, after cultivation on PDA medium for 12 days, the colony sizes of the Δ*mga*1, Δ*mga*2, Δ*mga*3 and Δ*mga*2+3 strains were similar to that of *M. ruber* M7, while those of Δ*mga*1+2 and Δ*mga*1+3 were about 45% and 80% smaller than *M. ruber* M7, respectively. We found that when a single Gα gene (*mga*1, *mga*2, or *mga*3) was deleted, the colony sizes did not significantly change. However, when the *mga*2 or *mga*3 gene was deleted in the Δ*mga*1 strain, the colony sizes were smaller than other mutants.

**FIGURE 2:**
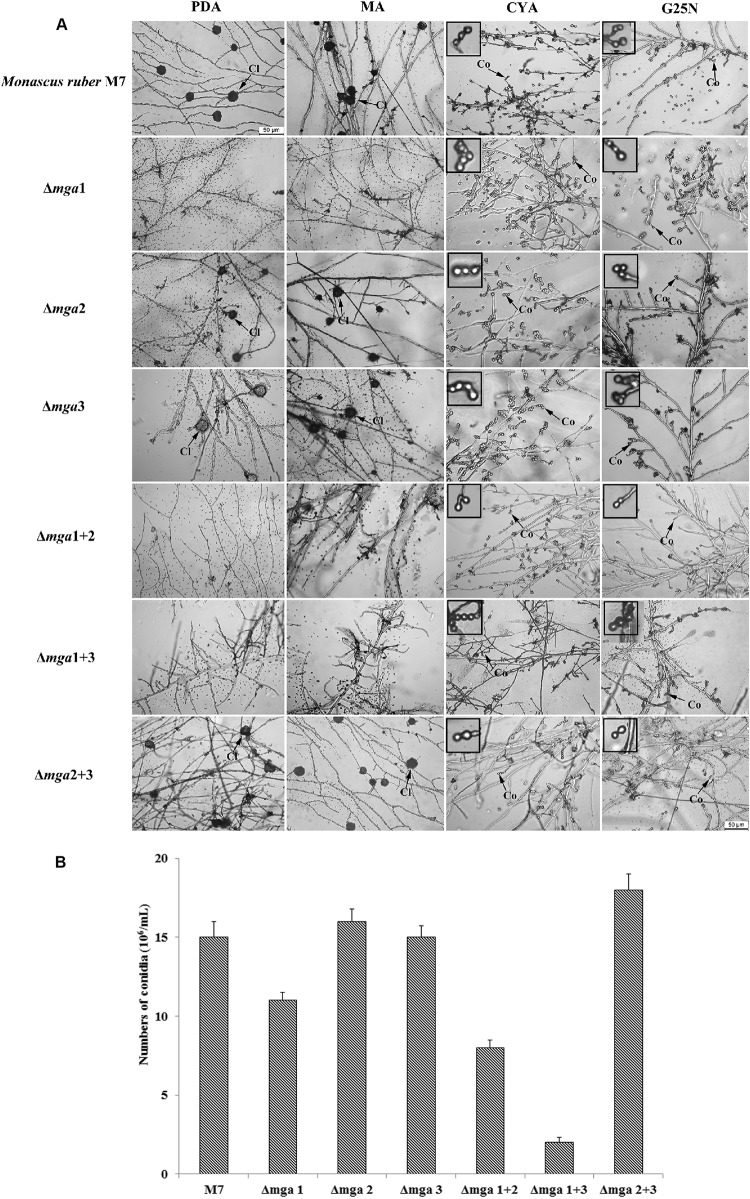
Microscopic structures of Gα mutants and *Monascus ruber* M7. **(A)** Cleistothecial (Cl) and conidial (Co) morphologies among M7 and Gα mutants were observed on PDA, MA, CYA, and G25N plates cultured at 28°C for 5 days. The enlarged areas are indicated by arrows. Size bar = 50 μm. **(B)** The numbers of conidia of the indicated strains were measured after growing on PDA medium at 28°C for 5 days.

Regarding sexual or asexual reproduction, as shown in Figure 2, cleistothecia were not found in *mga*1-related mutants (Δ*mga*1, Δ*mga*1+2, and Δ*mga*1+3), and their conidia-forming abilities were also reduced. However, the other mutants (Δ*mga*2, Δ*mga*3, and Δ*mga*2+3) showed no difference in sexual and asexual reproduction compared with *M. ruber* M7.

#### MPs and CIT Production

**FIGURE 3:**
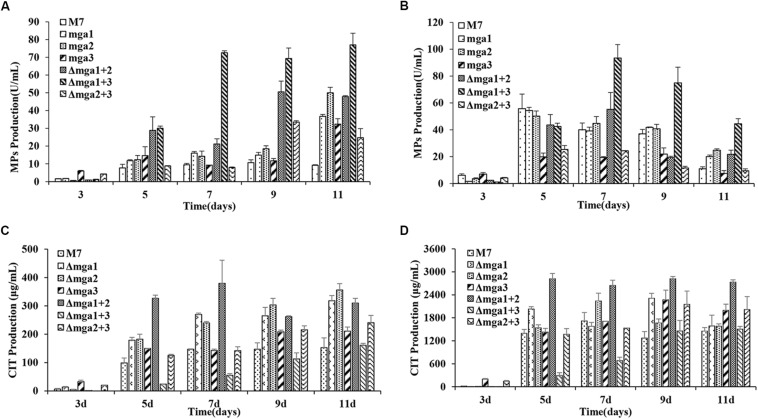
MPs and CIT levels in Gα mutants and *Monascus ruber* M7. **(A)** The intracellular MP production of Gα mutants and M7. **(B)** The extracellular MP production levels of Gα mutants and M7. **(C)** The intracellular CIT production levels of Gα mutants and M7. **(D)** The extracellular CIT production levels of Gα mutants and M7. The error bars indicate the standard deviations of three independent cultures. Significantly different at *P* < 0.01.

The intracellular and extracellular MPs and CIT production in *M. ruber* M7 and Gα mutants were analyzed (Figure 3). The intracellular MPs production levels in the Gα mutants, Δ*mga*1, Δ*mga*2, Δ*mga*3, Δ*mga*1+2, Δ*mga*1+3, and Δ*mga*2+3, were 2.1, 2.5, 1.9, 3.9, 6.5, and 2.1 times that of *M. ruber* M7, respectively. For extracellular MP production, only Δ*mga*1+3 was 1.7 times that of *M. ruber* M7, while the other five mutants possessed similar yields to *M. ruber* M7. For CIT, the single-deletion mutants and Δ*mga*1+2 had 1.2- to 2.2-fold increases in CIT production compared with *M. ruber* M7. Only during the later stage (9–11 d) the CIT production in Δ*mga*2+3 was greater (1.3-fold) than that in *M. ruber* M7. During the early stage (3–7 days) the CIT production in Δ*mga*1+3 was only 20–40% that of *M. ruber* M7.

### DEG Analysis, Annotation and Functional Classification

The RNAs of *M. ruber* M7 and six deletion mutants (Δ*mga*1, Δ*mga*2, Δ*mga*3, Δ*mga*1+2, Δ*mga*1+3, and Δ*mga*2+3) were independently extracted for a further transcriptomic analysis. The obtained clean sequence reads of the 14 samples were validated by qRT-PCR. In total, 11 genes in the *M. ruber* M7 genome were randomly selected for relative gene expression comparisons between M7 and Δ*mga* 1+3 strain, the selected genes are listed in Supplementary Table S2. As shown in Supplementary Figure S4, the relative expression levels of these 11 random genes had the same trends as in the RNA-Seq, which indicated that the transcriptome sequencing was reliable.

#### DEGs Analyses and Transcriptome Classification

The genes that possessed an expression differential multiple greater than 1, as well as a *Q*-value not greater than 0.001, were selected as DEGs. Compared with *M. ruber* M7, different mutants at different time points have diverse trends in their numbers of DEGs. There were greater numbers of DEGs in double-deletion strains than in single-deletion strains. In particular, in the Δ*mga*1+3 strain, 1,858 and 2,000 genes showed down-regulated expression levels at 3^rd^ day and 7^th^ day, respectively, which were much greater numbers than those in the corresponding single-deletion strains Δ*mga*1 and Δ*mga*3. This may explain the distinctive phenotype of the Δ*mga*1+3 strain (Figure 2).

A GO enrichment analysis of DEGs was performed. GO has three ontologies: molecular biological function, cellular component and biological process. For each ontology, the functional enrichment was determined. Compared with *M. ruber* M7, metabolic process possessed the most DEGs in all the mutants at both 3 and 7 days, and most genes in this GO ontology were down-regulated, such as in cellular process, cell part and catalytic activity. Among the KEGG pathways, the metabolic pathway possessed the most DEGs in all the mutants at both 3^rd^ and 7^th^ day, and most genes in this KEGG pathway were down-regulated, including those involved in meiosis–yeast, SMs biosynthesis and carbon metabolism.

#### Gα Genes Positively Regulate Vegetative Growth

The DEGs of carbon and nitrogen source metabolism are listed in Supplementary Table S3. The regulation of carbon source metabolism mostly focuses on the tricarboxylic acid cycle (TCA cycle), meanwhile many major facilitator superfamily (MFS) transporters were down-regulated. RNA-Seq results revealed that the absence of Gα subunits generally depressed the TCA cycle, especially reducing the biosynthesis of citric acid and succinyl CoA. The absence of both Mga1 and Mga3 regulated most genes in the TCA cycle. Data on the DEGs related to the TCA cycle are presented in Supplementary Figure S5. On the basis of the GO and KEGG analyses, we also analyzed the influence of different Gα genes on nitrogen metabolism. The expression levels of genes related to nitrogen metabolism mostly decreased in the mutants, with Δ*mga*1+2 and Δ*mga*1+3 possessing the greatest numbers of DEGs related to nitrogen metabolism. This indicates that all the Gα genes positively regulated nitrogen metabolism. Data on DEGs related to nitrogen source metabolism are presented in Supplementary Figure S6. The decreased expression of vegetative growth-related genes corresponded to the repressed colony sizes of the Δ*mga*1+2 and Δ*mga*1+3 strains (Figure 1).

#### Gα Genes Play Different Roles in Sexual and Asexual Reproduction

In filamentous fungi, the central regulatory pathway of conidiospore formation generally consists of *aba*A, *brl*A and *wet*A genes (Yu, 2006). The most reported sexual reproduction- related genes are mating type (*MAT*)-related genes (Varga et al., 2014). In addition, the *velvet* family genes are related to sexual/asexual reproduction (Yu et al., 2008; Liu Q. et al., 2016). The expression changes in all these genes as determined by the DEGs analysis are listed in Supplementary Figure S7.

Compared with *M. ruber* M7, the expression level of the cleistothecia-related gene *MAT*1-2 was decreased only in *mga*1-deleted strains (Δ*mga*1, Δ*mga*1+2, and Δ*mga*1+3), while their expression levels increased in Δ*mga*3 at 3 days. They were not changed in the Δ*mga*2 and Δ*mga*2+3 strains. This explained why cleistothecia were not found in Δ*mga*1, Δ*mga*1+2, and Δ*mga*1+3 strains (Figure 2), and it suggested that Mga1 positively regulates sexual reproduction while Mga2 and Mga3 have slight effect in sexual reproduction. However, most genes involved in conidial production, including the conidiospore formation genes *brl*A and *wet*A, had increased expression levels in the Gα mutants, except in Δ*mga*1+3. Only the expression levels of *velvet* regulators were decreased in almost all the mutants. This is different from the phenotypic analysis (Figure 2) that the conidia-forming ability was reduced in *mga*1-related mutants (Δ*mga*1, Δ*mga*1+2, and Δ*mga*1+3) but not in the other mutants.

#### Gα Genes Negatively Regulate MPs and CIT Biosynthesis

RNA-Seq results revealed that the expression levels of MPs biosynthetic genes (Chen et al., 2017), except *Mpig*L, were increased in all the mutants at 7^th^ day. However, these genes in Δ*mga*1+2 and Δ*mga*1+3 were up-regulated at 3^rd^ and 7^th^ day. In addition, in the *mga*1-deleted strains (Δ*mga*1, Δ*mga*1+2, and Δ*mga*1+3) more genes were up-regulated than those in the other mutants. The DEGs involved in MPs biosynthesis are shown in Supplementary Figure S8. This result matches the increased MPs yields in Gα mutants (Figure 3) and indicates that Gα negatively regulates MPs production by regulating the MPs biosynthetic gene cluster.

According to the RNA-Seq results, genes in the CIT gene cluster (He and Cox, 2016) showed different trends on different days. Gα subunits mainly regulated the expression of the CIT gene cluster at 3^rd^ day. Most genes in the CIT biosynthetic gene cluster were up-regulated in the single-deletion mutants and Δ*mga*1+2, and CIT production also increased in these mutants (Figure 3). In Δ*mga*1+3, although the *pksCT* gene was up-regulated, most other genes (*MRR*1–4 and *MRR*7–8) in the cluster were down-regulated, and the early stage (3–7 days) CIT production in the Δ*mga*1+3 strain was lower than that in *M. ruber* M7. In Δ*mga*2+3, only *pksCT* and *MRL2* were up-regulated, and only in the later stage (9–11 days) the CIT production was greater than that in *M. ruber* M7 (Figure 3). Data on DEGs involved in CIT biosynthesis are provided in Supplementary Figure S9. This result indicates that Gα genes (mainly *mga*1 and *mga*2) negatively influenced CIT production by regulating the CIT biosynthetic gene cluster.

## Conclusion and Discussion

G-protein signaling pathways play important roles in fungal reproduction and SMs production, and the functions of different Gα subunits (Gα1–3) have been analyzed in some fungi using single gene modification (Xu et al., 2015; Yoda et al., 2015; Zhang et al., 2016). The positively regulatory function of the Gα1 subunit on colony growth and asexual reproduction, which is conserved and extensive in most reported fungi, has been extensively researched (Li et al., 2010; Yang et al., 2012; Hu et al., 2013; Wasil et al., 2013; Garcia-Rico et al., 2017). However, until now, there has been no literature regarding double deletions combined with RNA-Seq of Gα subunit genes. In the current study, single- and double-gene(s) deletion mutants of the three Gα subunits were first systematically analyzed to determine the effects of different Gα subunits on *M. ruber* M7 according to the phenotypic characteristics combined with RNA-Seq analyses. The results show that all three Gα subunits (Mga1-3) in *M. ruber* M7 work together to regulate biological processes. Briefly, Mga1 comprehensively regulates the growth, development and secondary metabolism, while Mga2 and Mga3 act as supplementary regulators on growth and secondary metabolism. These findings not only illuminate the functions of different Gα subunits in *M. ruber* M7, but also provide a deeper understanding of the functional connections among different Gα subunits that involve regulating growth, development and secondary metabolism in other filamentous fungi.

Different Gα subunits (Gα1–3) regulate different biological processes in fungi (Li et al., 2010; Yang et al., 2012; Hu et al., 2013; Wasil et al., 2013; Garcia-Rico et al., 2017). For vegetative growth, Gα1 positively regulate the related processes in fungi such as *Penicillium camembertii* and *Fusarium oxysporum* (Guo et al., 2016b; Garcia-Rico et al., 2017), and Gα2 has no significant influence on fungal vegetative growth in *Valsa mali* and *F. oxysporum* (Guo et al., 2016a; Song et al., 2017), while Gα3 possesses different regulatory functions in different fungi. For example, PGA3 (Gα3) in *P. camembertii* and Gvm3 (Gα3) in *V. mali* positively regulate vegetative growth (Hu et al., 2013; Song et al., 2017), while FGA3 (Gα3) in *F. oxysporum* has no influence on vegetative growth (Guo et al., 2016b). In the current study, we find that Mga1(Gα1) has slightly effects on the vegetative growth of *M. ruber* M7, while Mga2 and Mga3 have no significant effects, which is similar to the results in *P. camembertii* and *F. oxysporum* (Guo et al., 2016b; Garcia-Rico et al., 2017), and the colony sizes of Δ*mga*1+2 and Δ*mga*1+3 are much smaller than those of *M. ruber* M7 and Δ*mga*1, which suggests that the Mga2 and Mga3 subunits enhance this regulatory process when either is deleted along with Mga1. In addition, a group of MFS transporters involved in carbon source metabolism are more down-regulated in the Δ*mga*1+2 and Δ*mga*1+3 strains than those in *M. ruber* M7 according to RNA-Seq analyses (Supplementary Table S3), which implies that the transportation of carbon sources may be essential for *Monascus* growth and that Gα subunits may directly regulate MFS transporters to affect *Monascus* vegetative growth. Thus, further investigations of these transporters could contribute to determining the key elements involved in *Monascus* and other fungi vegetative growth.

Asexual reproduction, in many filamentous fungi, is mainly positively regulated by the sporogenesis central regulatory genes, including *aba*A, *brl*A and *wet*A (de Vries et al., 2017; Wu et al., 2018). However, in this study, the increased expression levels of *brl*A and *wet*A (no *aba*A in *Monascus* genome) in *mga*1-related mutants (Δ*mga*1, Δ*mga*1+2, and Δ*mga*1+3) do not enhance conidial reproduction. This implies that a new asexual reproduction-related regulatory pathway might exist in *M. ruber* M7. Further studies on reproduction related regulatory pathways which we are doing, might find a new asexual reproduction regulatory pathway in *Monascus* spp.

The Gα regulation of SMs biosynthesis has been verified by single gene deletions, indicating that the negative regulation of Gα1 is conserved in most fungi (Yu et al., 2008; Guo et al., 2016a), and Gα2’s regulatory roles are diverse. For example, Gvm2 (Gα2) in *V. mali* negatively regulates SMs biosynthesis (Song et al., 2017), while GanA (Gα2) in *Aspergillus nidulans* has no influence on SMs biosynthesis (Yu, 2006). Additionally, Gα3 has no significant influence on SMs biosynthesis (Chang et al., 2004; Guo et al., 2016b). In our study, the single gene deletions have revealed that Mga1 (Gα1) and Mga2 (Gα2) can negatively regulate MPs and CIT production and that Mga3 (Gα3) has no significant effect. These results are similar to those of studies in *V. mali* and *F. oxysporum* (Guo et al., 2016b; Song et al., 2017). Moreover, double-gene deletions of Gα1–3 subunits can jointly regulate SMs. For instance, Mga2 and Mga3 combined with Mga1 can negatively regulate MPs production, since according to the phenotypic and transcriptomics analyses, Δ*mga*1+2 and Δ*mga*1+3 strains have much greater MPs yields (Figure 3) as well as greater numbers of up-regulated MPs biosynthesis-related DEGs compared with the other mutants (Supplementary Figure S8).

The RNA-Seq results (Supplementary Table S4) show that, besides MPs and CIT polyketide synthase (PKS) genes, many other PKS and non-ribosomal peptide synthetase genes are also regulated by Gα subunits. This is especially true of the Δ*mga*1+3 strain in which nearly all the PKS and non-ribosomal peptide synthetase genes are differentially expressed. The analyses of related SMs in Δ*mga*1+3 may help to improve our understanding of *Monascus* SMs.

**FIGURE 4:**
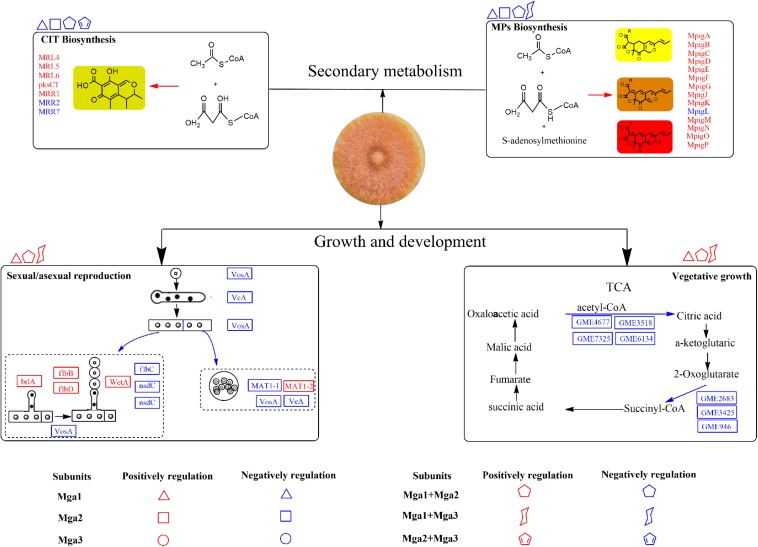
The possible gene regulatory network of Gα subunits in *Monascus*. The proteins and arrows marked in red indicate that they are up-regulated, the proteins and arrows marked in blue indicate that they are down-regulated.

Based on the above findings, a Gα regulatory system in *M. ruber* M7 is proposed in Figure 4. First, vegetative growth is mainly positively regulated by Mga1, and Mga2 and Mga3 can improve this regulatory process when either is deleted along with Mga1. All the Gα subunits positively regulate carbon and nitrogen metabolism (Supplementary Table S3) to affect vegetative growth. Second, Mga1 contributes the most to the regulation of sexual/asexual reproduction compared with Mga2 and Mga3, and the regulation of asexual reproduction may occur prior to the central regulatory pathway. The regulation of sexual reproduction is reflected in the regulation of *MAT*1-2 gene, which is down-regulated in *mga*1-deleted strains (Supplementary Figure S7). Third, Gα subunits in *M. ruber* M7 negatively regulate the SMs. In detail, Mga1 and Mga2 can negatively regulate MPs and CIT production individually or jointly, while Mga3 may combine with Mga1 to only negatively regulate MPs yields.

## Data Availability

The raw data supporting the conclusions of this manuscript will be made available by the authors, without undue reservation, to any qualified researcher.

## Author Contributions

FC managed the project. ML, JL, YS, and YF conducted the transformants construction, secondary metabolites analysis, and transcriptome results analysis in this work. LL constructed the Δ*mga*1 strain. ML conducted the phenotypic characterization, and interpreted the analysis results and wrote the manuscript. J-HY and YF contributed to the revision of the manuscript. All authors reviewed the manuscript.

## Conflict of Interest Statement

The authors declare that the research was conducted in the absence of any commercial or financial relationships that could be construed as a potential conflict of interest.

## Footnotes

1 http://en.genomics.cn/

2 http://www.geneontology.org/

3 https://www.kegg.jp/

## Supplementary Material

FIGURE S1 Deletion strategy and confirmation of Δ*mga*2 and Δ*mga*3 mutants. **(A)** The strategy to construct the Δ*mga*2 strain and *mga*2 complementary strain (R*mga*2). **(B)** The strategy to construct the Δ*mga*3 strain and *mga*3 complementary strain (R*mga*3). **(C)** PCR identification of Δ*mga*2. PCR products of M7, Δ*mga*2, and R*mga*2 with different primers; M, *Trans* 2K plus II marker. **(D)** PCR identification of Δ*mga*3. PCR products of M7, Δ*mga*3, and R*mga*3 with different primers; M, *Trans* 2K plus II marker. **(E)** Southern hybridization analysis. *Sac*I and *Xho*I are used in double-digesting genomic DNA, M: λDNA/*Hin*dIII marker. Probe 1, *mga*2 ORF; Probe 2, *hph* gene; Probe 3, *mga*3 ORF; Probe 4, *neo* gene.

FIGURE S2 Deletion strategy and confirmation of double-deletion strains. **(A)** The strategy to construct the Δ*mga*1+2 strain. **(B)** The strategy to construct the Δ*mga*1+3 and Δ*mga*2+3 strains. **(C)** PCR identification of double-deletion strains; M, *Trans* 2K plus II marker. **(D)** Southern hybridization analysis. *Kpn*I is used in digesting genomic DNA; M, λDNA/*Hin*dIII marker; Probe 1, *mga*2 ORF; Probe 3, *mga*3 ORF; Probe 4, *neo* gene.

FIGURE S3 Colony morphologies and microscopic structures of R*mga*2 (Δ*mga*2::*mga*2) and R*mga*3 (Δ*mga*3::*mga*3) strains. **(A)** Colony morphologies of M7, Δ*mga*2 strain and R*mga*2 mutants observed on PDA, MA, CYA, and G25N plates and cultured at 28°C for 12 days. **(B)** Cl and Co morphologies among M7, Δ*mga*2 strain and R*mga*2 mutants were observed on PDA, MA, CYA, and G25N plates cultured at 28°C for 5 days. The enlarged areas are indicated by arrows. Size bar = 100 μm. **(C)** Colony morphologies of M7, Δ*mga*3 strain and R*mga*3 mutants observed on PDA, MA, CYA, and G25N plates and cultured at 28°C for 12 days. **(D)** Cl and Co morphologies among M7, Δ*mga*3 strain and R*mga*3 mutants were observed on PDA, MA, CYA, and G25N plates cultured at 28°C for 5 days. The enlarged areas are indicated by arrows. Size bar = 50 μm.

FIGURE S4 Gene expression levels analyzed by RNA-Seq and qRT-PCR. The *x*-axis represents the selected 11 genes; the *y*-axis on the left side represents the gene expression levels as assessed by RNA-Seq; the *y*-axis on the right side represents the relative gene expression level as assessed by qRT-PCR.

FIGURE S5 The influence of Gα mutants on the TCA cycle. Blue indicates decreased enzyme expression; red indicates increased enzyme expression.

FIGURE S6 The DEGs involved in the nitrogen metabolism of Gα mutants. Blue indicates down-regulation in the Gα mutants; red indicates up-regulation in the Gα mutants; gray indicates not regulated in the Gα mutants.

FIGURE S7 The DEGs involved in the sporulation of Gα mutants. Blue indicates down-regulation in the Gα mutants; red indicates up-regulation in the Gα mutants; gray indicates not regulated in the Gα mutants.

FIGURE S8 The DEGs involved in the MP biosynthesis of Gα mutants. Blue indicates down-regulation in the Gα mutants; red indicates up-regulation in the Gα mutants; gray indicates not regulated in the Gα mutants.

FIGURE S9 The DEGs involved in the CIT biosynthesis of Gα mutants. Blue indicates down-regulation in the Gα mutants; red indicates up-regulation in the Gα mutants; gray indicates regulated in the Gα mutants.
