# Supplementary figures and images for: Effects of Different G-Protein α-Subunits on Growth, Development and Secondary Metabolism of Monascus ruber M7
Source: Front Microbiol. 2019 Jul 9;10:1555. doi: 10.3389/fmicb.2019.01555 (PMC6632705; doi:10.3389/fmicb.2019.01555)

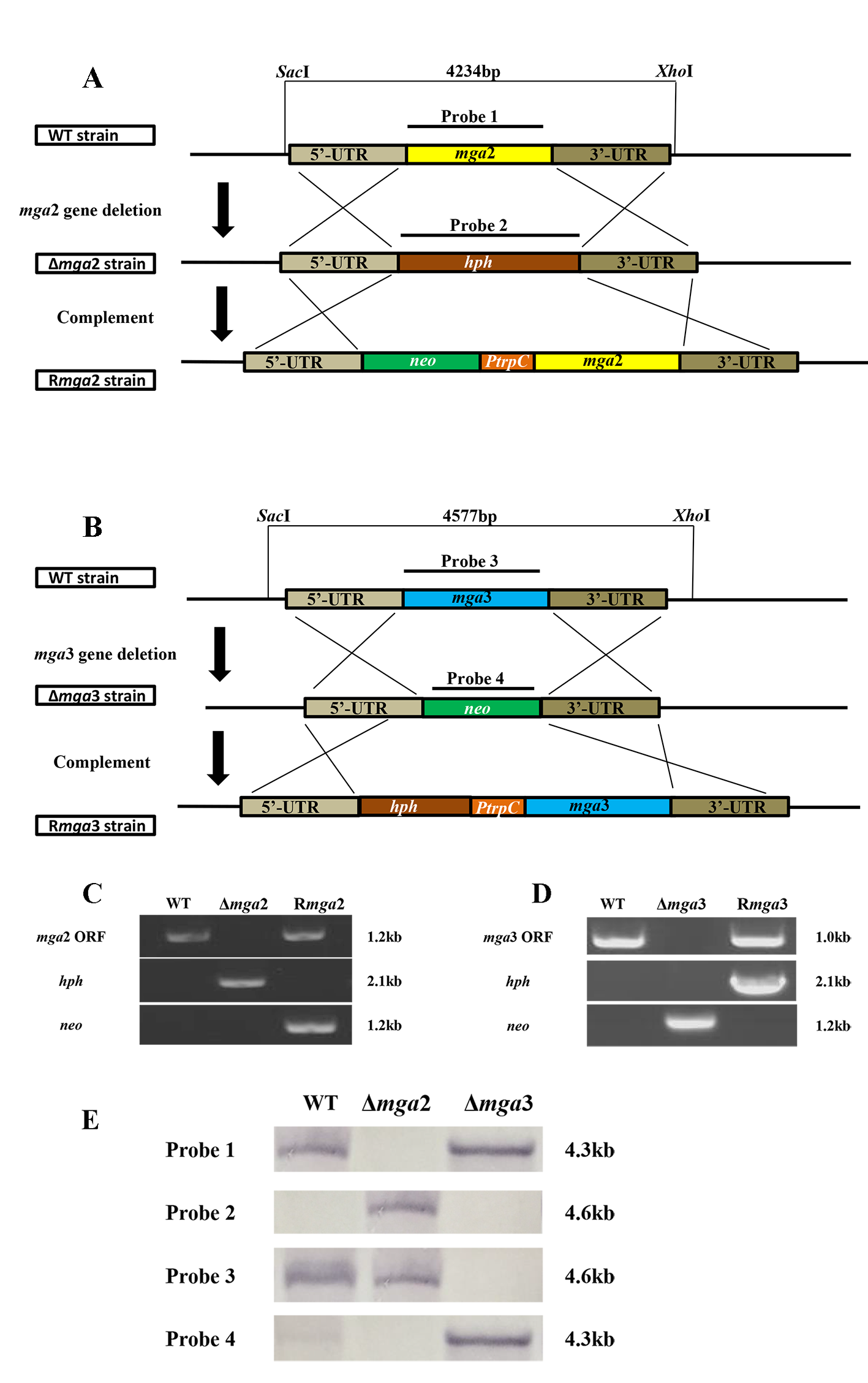

Supplement: FIGURE S1 — Deletion strategy and confirmation of Δmga2 and Δmga3 mutants. (A) The strategy to construct the Δmga2 strain and mga2 complementary strain (Rmga2). (B) The strategy to construct the Δmga3 strain and mga3 complementary strain (Rmga3). (C) PCR identification of Δmga2. PCR products of M7, Δmga2, and Rmga2 with different primers; M, Trans 2K plus II marker. (D) PCR identification of Δmga3. PCR products of M7, Δmga3, and Rmga3 with different primers; M, Trans 2K plus II marker. (E) Southern hybridization analysis. SacI and XhoI are used in double-digesting genomic DNA, M: λDNA/HindIII marker. Probe 1, mga2 ORF; Probe 2, hph gene; Probe 3, mga3 ORF; Probe 4, neo gene. [file Image_1.TIF]

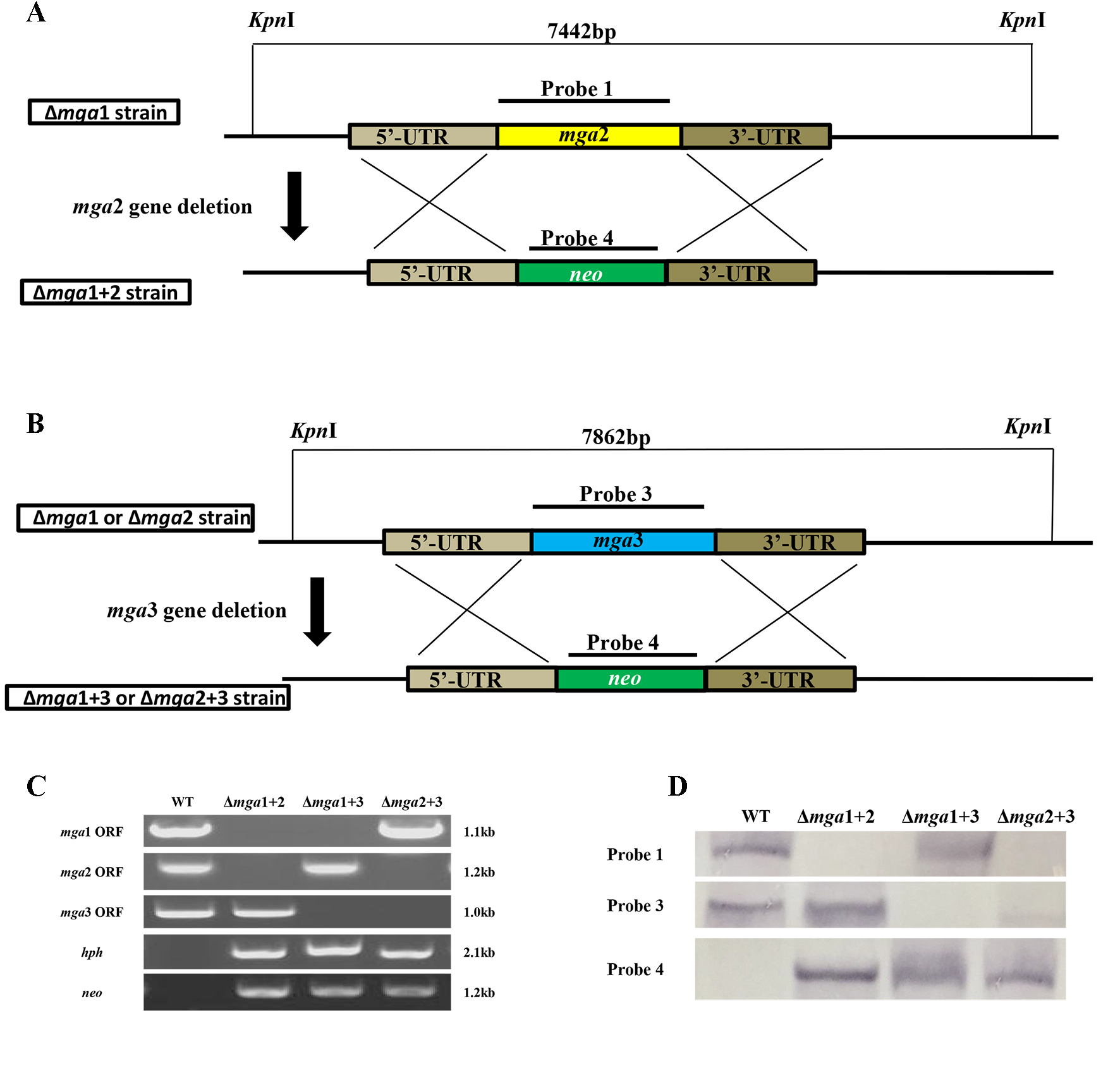

Supplement: FIGURE S2 — Deletion strategy and confirmation of double-deletion strains. (A) The strategy to construct the Δmga1+2 strain. (B) The strategy to construct the Δmga1+3 and Δmga2+3 strains. (C) PCR identification of double-deletion strains; M, Trans 2K plus II marker. (D) Southern hybridization analysis. KpnI is used in digesting genomic DNA; M, λDNA/HindIII marker; Probe 1, mga2 ORF; Probe 3, mga3 ORF; Probe 4, neo gene. [file Image_2.TIF]

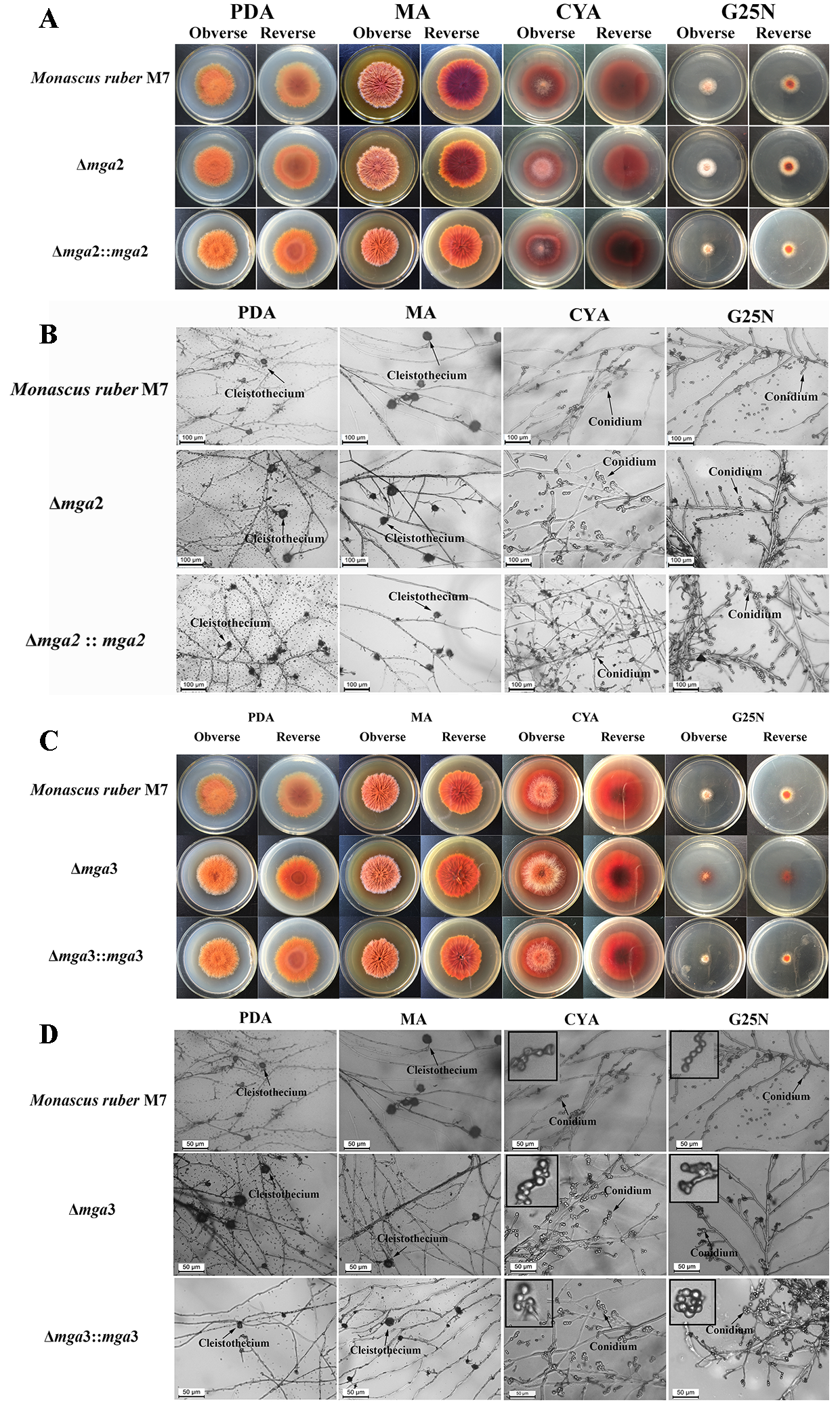

Supplement: FIGURE S3 — Colony morphologies and microscopic structures of Rmga2 (Δmga2::mga2) and Rmga3 (Δmga3::mga3) strains. (A) Colony morphologies of M7, Δmga2 strain and Rmga2 mutants observed on PDA, MA, CYA, and G25N plates and cultured at 28°C for 12 days. (B) Cl and Co morphologies among M7, Δmga2 strain and Rmga2 mutants were observed on PDA, MA, CYA, and G25N plates cultured at 28°C for 5 days. The enlarged areas are indicated by arrows. Size bar = 100 μm. (C) Colony morphologies of M7, Δmga3 strain and Rmga3 mutants observed on PDA, MA, CYA, and G25N plates and cultured at 28°C for 12 days. (D) Cl and Co morphologies among M7, Δmga3 strain and Rmga3 mutants were observed on PDA, MA, CYA, and G25N plates cultured at 28°C for 5 days. The enlarged areas are indicated by arrows. Size bar = 50 μm. [file Image_3.TIF]

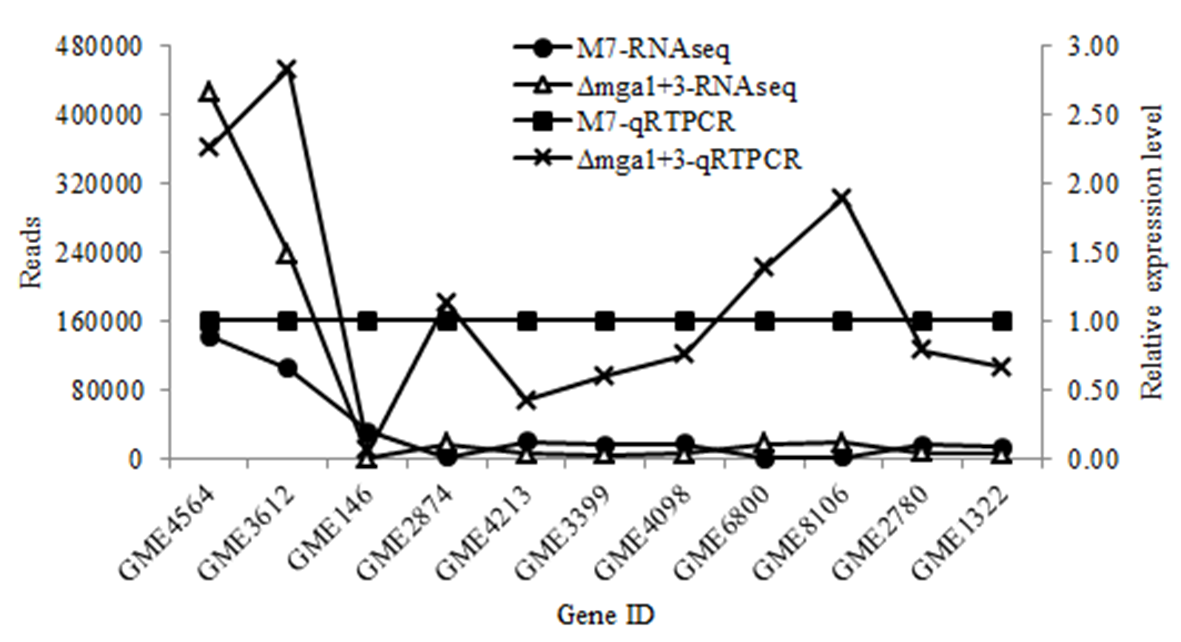

Supplement: FIGURE S4 — Gene expression levels analyzed by RNA-Seq and qRT-PCR. The x-axis represents the selected 11 genes; the y-axis on the left side represents the gene expression levels as assessed by RNA-Seq; the y-axis on the right side represents the relative gene expression level as assessed by qRT-PCR. [file Image_4.TIF]

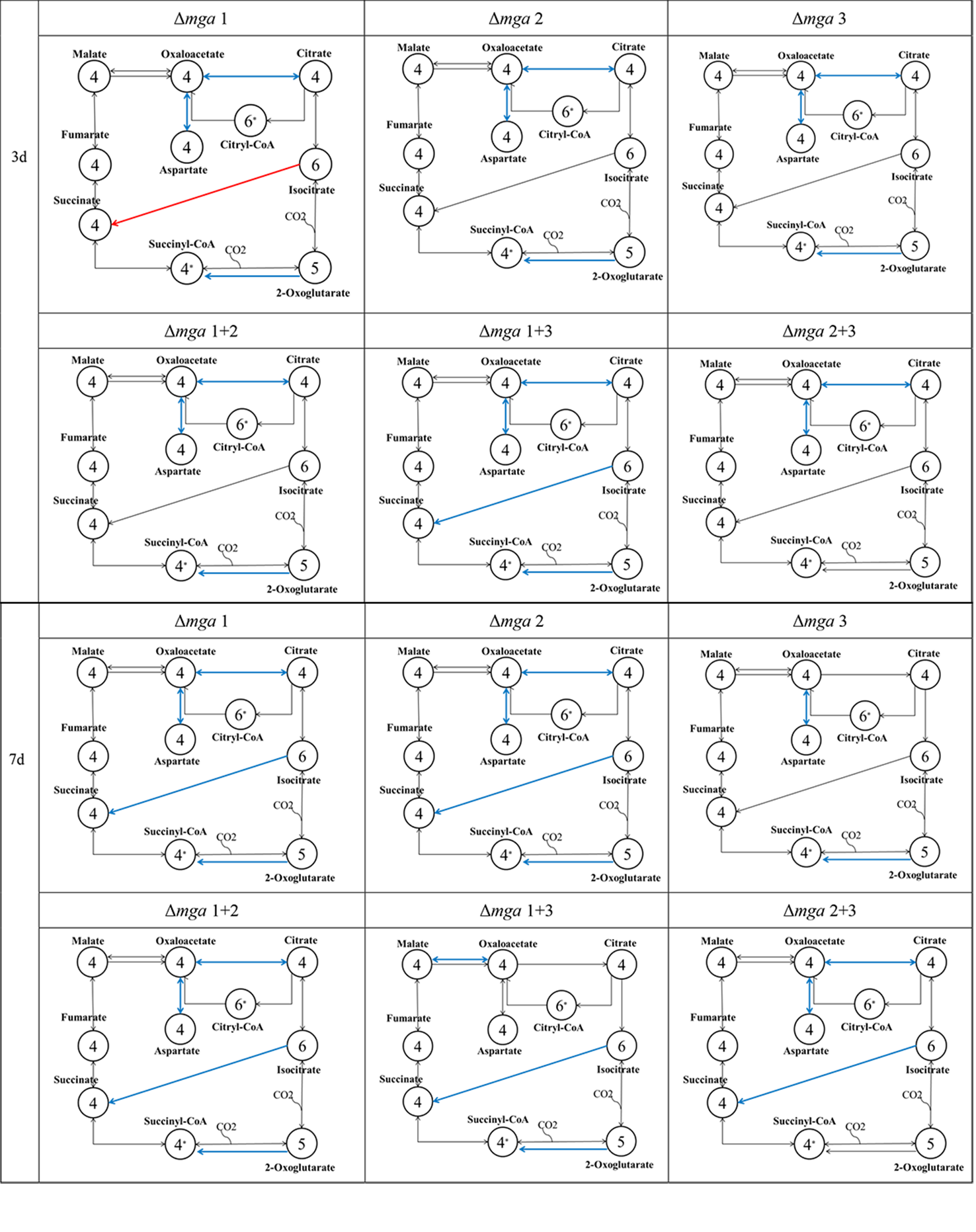

Supplement: FIGURE S5 — The influence of Gα mutants on the TCA cycle. Blue indicates decreased enzyme expression; red indicates increased enzyme expression. [file Image_5.TIF]

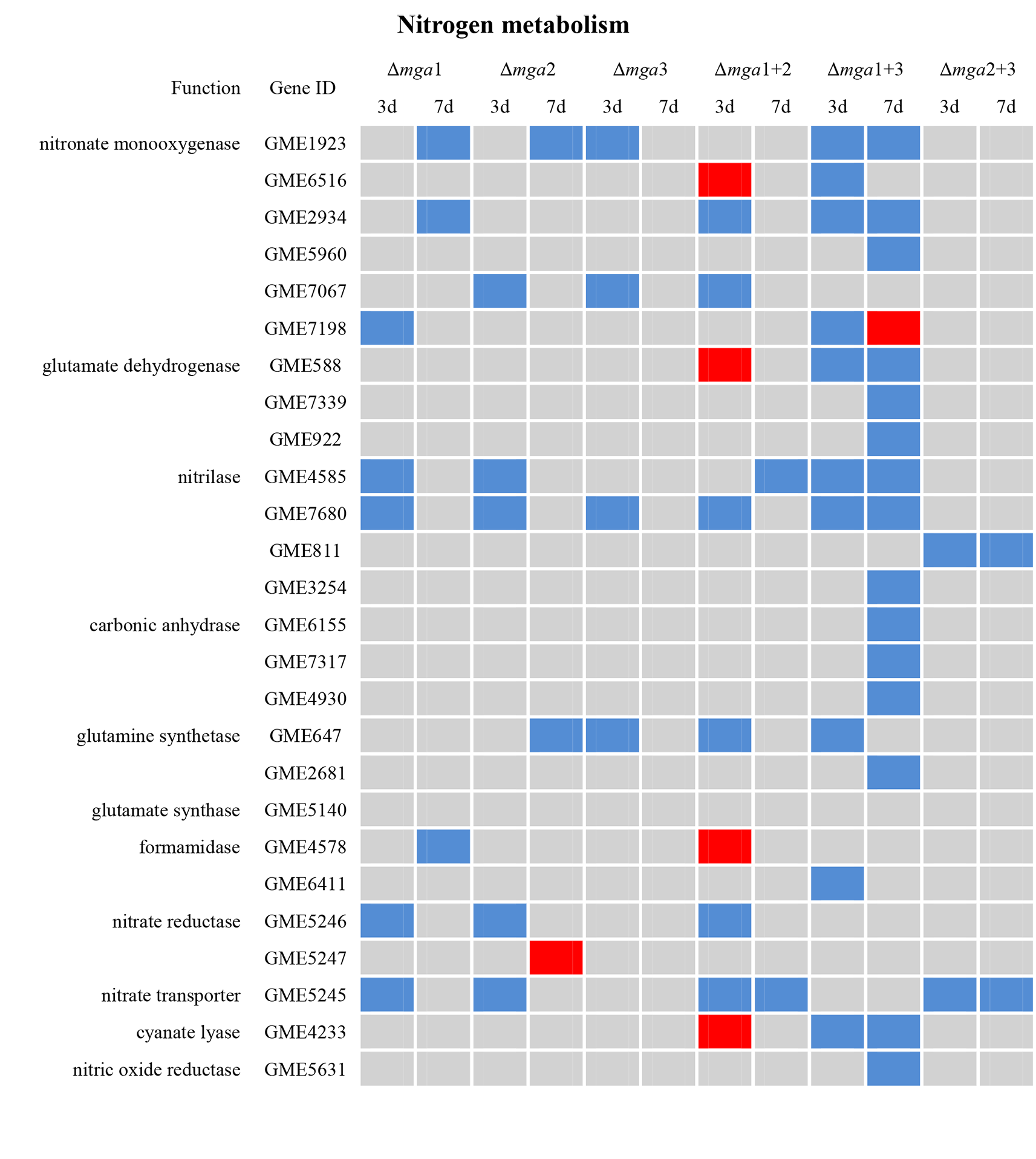

Supplement: FIGURE S6 — The DEGs involved in the nitrogen metabolism of Gα mutants. Blue indicates down-regulation in the Gα mutants; red indicates up-regulation in the Gα mutants; gray indicates not regulated in the Gα mutants. [file Image_6.TIF]

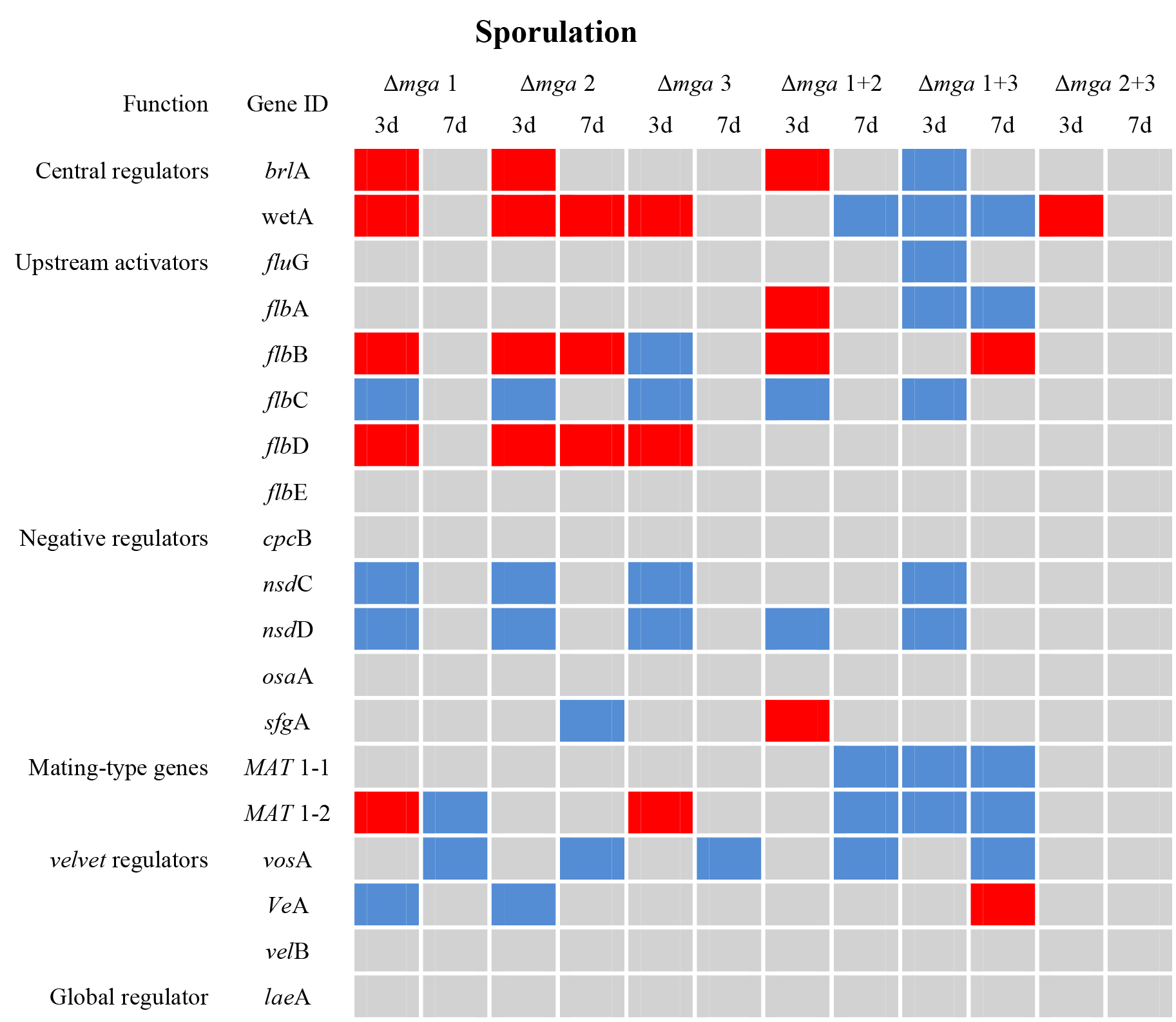

Supplement: FIGURE S7 — The DEGs involved in the sporulation of Gα mutants. Blue indicates down-regulation in the Gα mutants; red indicates up-regulation in the Gα mutants; gray indicates not regulated in the Gα mutants. [file Image_7.TIF]

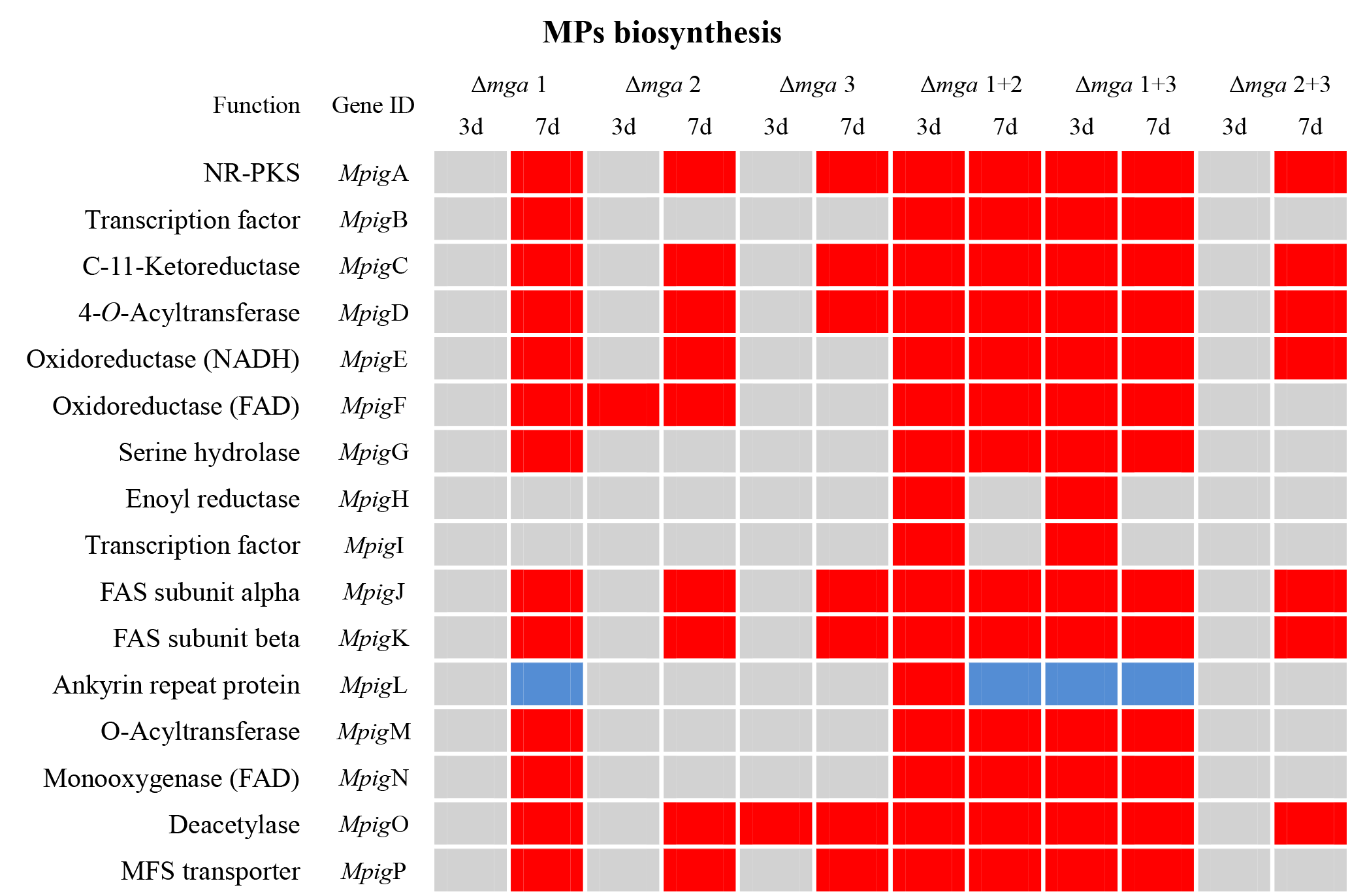

Supplement: FIGURE S8 — The DEGs involved in the MP biosynthesis of Gα mutants. Blue indicates down-regulation in the Gα mutants; red indicates up-regulation in the Gα mutants; gray indicates not regulated in the Gα mutants. [file Image_8.TIF]

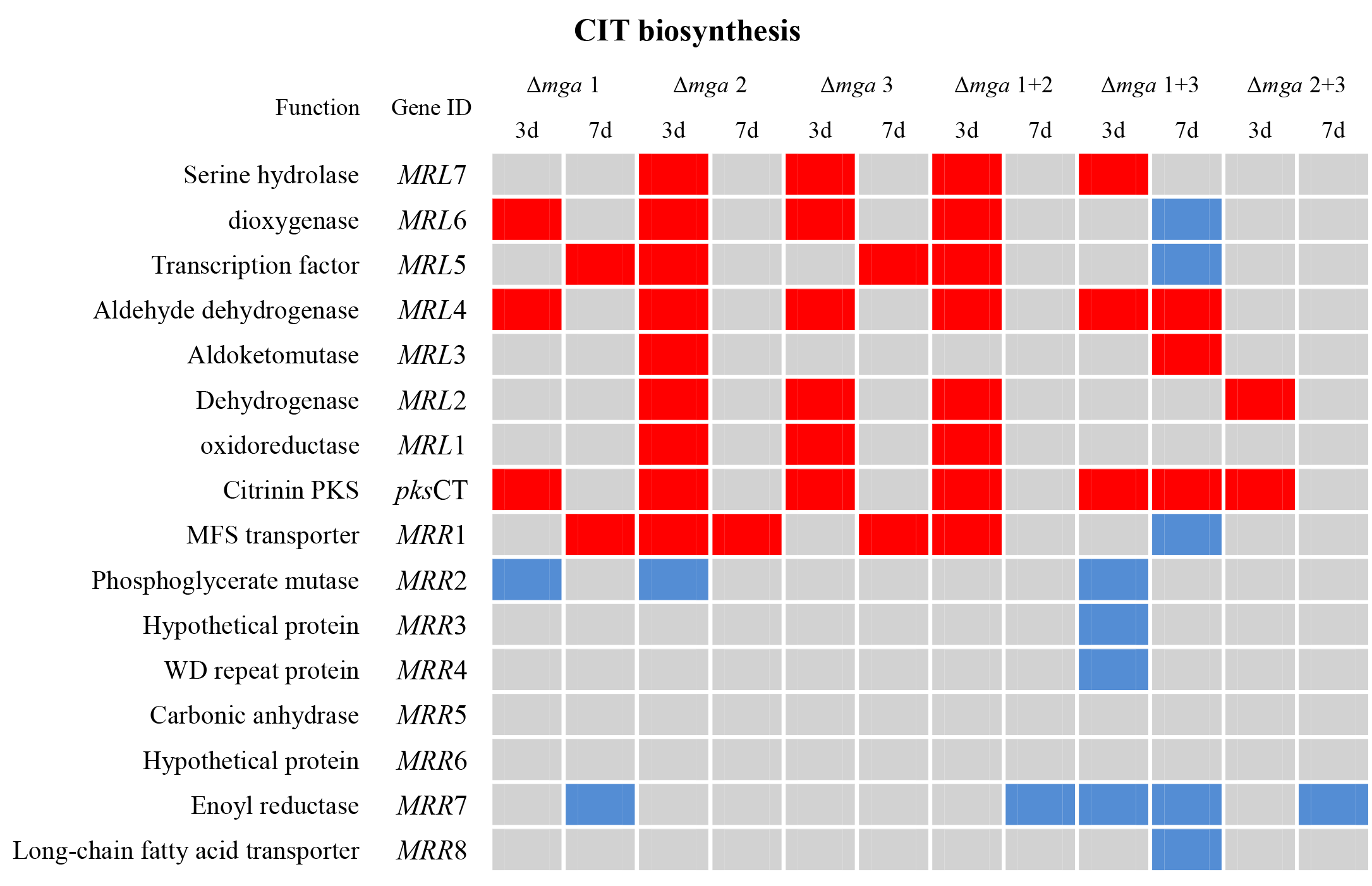

Supplement: FIGURE S9 — The DEGs involved in the CIT biosynthesis of Gα mutants. Blue indicates down-regulation in the Gα mutants; red indicates up-regulation in the Gα mutants; gray indicates regulated in the Gα mutants. [file Image_9.TIF]
