# Supplementary material for: Effects of Different G-Protein α-Subunits on Growth, Development and Secondary Metabolism of Monascus ruber M7
Source: Front Microbiol. 2019 Jul 9;10:1555. doi: 10.3389/fmicb.2019.01555 (PMC6632705; doi:10.3389/fmicb.2019.01555)
Supplement: Supplementary file 10 [file Table_1.DOCX]

**Table S1 Primers for deletion of Gα gene**

| **Primers** | **Sequences (5’→3’)** | **Functions** |
| --- | --- | --- |
| mga2-5up | CCGCTCGAGTTGATTGTTGCTCTCCTCAT | Amplified the 5’ homologous sequence to construct the Δ*mga2* strain, length 1086 bp |
| mga2-5do | CTCCTTCAATATCATCTTCTGTCGACCTGGGCACTTTAGGTAGTTC |  |
| mga2-3up | GTTTAGAGGTAATCCTTCTTTCTAGATGAGAAACTGGCGGTGGTG | Amplified the 3’ homologous sequence to construct the Δ*mga2* strain, length 1011 bp |
| mga2-3do | GCGGATCACCACCAGGATATGT |  |
| mga2-O-up | GGAAGCCGCAAAAGCCTGAC | Amplified *mga*2 orf，**Probe 1** for Southern hybridization, length 1257 bp |
| mga2-O-do | CCGCACCCGAGAATAGAATC |  |
| hph-up | GTCGACAGAAGATGATATTG | Amplified *hph* sequence, **Probe 2** for Southern hybridization, length 2137 bp |
| hph-do | CTAGAAAGAAGGATTACCTC |  |
| mga3-5up | ATGTTCGCACCAGTACAACT | Amplified the 5’ homologous sequence to construct the Δ*mga*3 strain, length 1132bp |
| mga3-5do | GTTCGATGGGGTTGAGTTGGTTTACCCTTCGGAATC |  |
| mga3-3up | CATGCATGTTGCATGATGATTCTTTCCTCCCGTTTG | Amplified the 3’ homologous sequence to construct the Δ*mga*3 strain, length 1209 bp |
| mga3-3do | ACAGGGCGGGTACATT |  |
| mga3-O-up | TCTCGGTCTGTCTGTCTGTT | Amplified *mga*3 orf, **Probe 3** for Southern hybridization, length 1045 bp |
| mga3-O-do | GAAGGATGGAGAGAAGACAT |  |
| G418-up | CCAACTCAACCCCATCGAACCGTAACC | Amplified *neo* sequence, **Probe 4** for Southern hybridization, length 1221 bp |
| G418-do | ATCATCATGCAACATGCATG |  |
| mga2-neo-5up | CCCAGCAACCTAGAC | Amplified the 5’ homologous sequence to construct the Δ*mga*1+2 strain, length 1210 bp |
| mga2-neo-5do | GTTCGATGGGGTTGAGTTGGAACCAATCCCAATAAC |  |
| mga2-neo-3up | CATGCATGTTGCATGATGATCGGAAAGGAGCTGTAGGAT | Amplified the 3’ homologous sequence to construct the Δ*mga*1+2 strain, length 1011 bp |
| mga2-neo-3do | GGAGAAGACGGGACAACTAG |  |
