# Supplementary material for: Effects of Different G-Protein α-Subunits on Growth, Development and Secondary Metabolism of Monascus ruber M7
Source: Front Microbiol. 2019 Jul 9;10:1555. doi: 10.3389/fmicb.2019.01555 (PMC6632705; doi:10.3389/fmicb.2019.01555)
Supplement: Supplementary file 11 [file Table_2.DOCX]

**Table S2 Genes selected to run qRT-PCR**

| Gene ID | Gene function |  | Gene ID | Gene function |
| --- | --- | --- | --- | --- |
| GME4564 | acetyltransferase |  | GME4098 | GPI anchored protein |
| GME3612 | aldehyde dehydrogenase | | GME6800 | NAD(P)H dehydrogenase |
| GME146 | acid phosphatase |  | GME8106 | kynurenine aminotransferase |
| GME2874 | trichodiene synthase | | GME2780 | uncharacterized UPF0442 protein |
| GME4213 | hypothetical protein | | GME1322 | magnesium ion transporter |
| GME3399 | hypothetical protein | |  |  |
