# Supplementary material for: Effects of Different G-Protein α-Subunits on Growth, Development and Secondary Metabolism of Monascus ruber M7
Source: Front Microbiol. 2019 Jul 9;10:1555. doi: 10.3389/fmicb.2019.01555 (PMC6632705; doi:10.3389/fmicb.2019.01555)
Supplement: Supplementary file 12 [file Table_3.DOCX]

**Table S3 The DEGs of carbon and nitrogen source metabolism**

| Gene ID | Function |  |  | Gene ID | Function |  |
| --- | --- | --- | --- | --- | --- | --- |
| GME2683 | MFS transporter | down |  | GME1923 | nitronate monooxygenase | down |
| GME3425 | MFS transporter | down |  | GME2934 | nitronate monooxygenase | down |
| GME3518 | MFS transporter | down |  | GME5960 | nitronate monooxygenase | down |
| GME3986 | MFS transporter | down |  | GME6516 | nitronate monooxygenase | up |
| GME4542 | MFS transporter | down |  | GME7067 | nitronate monooxygenase | down |
| GME4677 | MFS transporter | down |  | GME7198 | nitronate monooxygenase | down |
| GME5810 | MFS transporter | down |  | GME4930 | carbonic anhydrase | down |
| GME5915 | MFS transporter | down |  | GME6155 | carbonic anhydrase | down |
| GME6000 | MFS transporter | down |  | GME7317 | carbonic anhydrase | down |
| GME6134 | MFS transporter | down |  | GME3254 | nitrilase | down |
| GME946 | MFS transporter | down |  | GME4585 | nitrilase | down |
| GME195 | aspartate aminotransferase | down |  | GME7680 | nitrilase | down |
| GME2065 | aspartate aminotransferase | down |  | GME811 | nitrilase | down |
| GME5651 | aspartate aminotransferase | down |  | GME588 | glutamate dehydrogenase | up |
| GME5679 | aspartate aminotransferase | up |  | GME7339 | glutamate dehydrogenase | down |
| GME5875 | aspartate aminotransferase | down |  | GME922 | glutamate dehydrogenase | down |
| GME841 | aspartate aminotransferase | down |  | GME1452 | citrate synthase | up |
| GME4482 | dihydroxyacetone synthase | up |  | GME361 | citrate synthase | up |
| GME6656 | dihydroxyacetone synthase | down |  | GME7325 | citrate synthase | down |
| GME7090 | ribose 5-phosphate isomerase | down |  | GME6614 | isocitrate dehydrogenase | up |
| GME3661 | ribose-5-phosphate isomerase | down |  | GME6665 | isocitrate dehydrogenase | up |
| GME3174 | ribose-phosphate pyrophosphokinase | up |  | GME5250 | isocitrate dehydrogenase | up |
| GME4628 | ribose-phosphate pyrophosphokinase | down |  | GME2681 | glutamine synthetase | up |
| GME3765 | succinate dehydrogenase | up |  | GME647 | glutamine synthetase | down |
| GME464 | succinate dehydrogenase | up |  | GME4578 | formamidase | down |
| GME4233 | cyanate lyase | down |  | GME6411 | formamidase | down |
| GME6344 | transketolase | down |  | GME5246 | nitrate reductase | up |
| GME8117 | 2-oxoglutarate dehydrogenase | down |  | GME5245 | nitrate transporter CrnA | down |
| GME5880 | 6-phosphogluconate dehydrogenase | down |  | GME5631 | nitric oxide reductase | down |
| GME463 | fructose-bisphosphate aldolase | up |  | GME5247 | nitrite reductase | up |

Significantly different expression was identified by NOISeq method with an absolute value of log_2_-fold change >1 and Probability>0.8.

“Up” means an increased expression over M7; “Down” means a decrease expression over M7.
