# Supplementary material for: Effects of Different G-Protein α-Subunits on Growth, Development and Secondary Metabolism of Monascus ruber M7
Source: Front Microbiol. 2019 Jul 9;10:1555. doi: 10.3389/fmicb.2019.01555 (PMC6632705; doi:10.3389/fmicb.2019.01555)
Supplement: Supplementary file 13 [file Table_4.DOCX]

**Table S4 The DEGs of 8 PKS and 5 NPRS in Gα-deletion mutants**

| Gene ID | Function | Δ*mga* 1 | | Δ*mga* 2 | | Δ*mga* 3 | | Δ*mga* 1+2 | | Δ*mga* 1+3 | | Δ*mga* 2+3 | |
| --- | --- | --- | --- | --- | --- | --- | --- | --- | --- | --- | --- | --- | --- |
|  |  | 3d^1*^ | 7d^2*^ | 3d | 7d | 3d | 7d | 3d | 7d | 3d | 7d | 3d | 7d |
| GME1661 | conidial yellow pigment PKS | — | — | — | ↓ | — | — | ↑ | — | ↓ | ↓ | — | — |
| GME2523 | lovastatin diketide synthase | — | ↓ | — | ↓ | — | ↓ | — | ↓ | ↓ | ↓ | — | ↓ |
| GME2757 | CIT PKS | ↑ | — | ↑ | — | ↑ | — | ↑ | — | ↑ | ↑ | ↑ | — |
| GME4561 | MPs PKS | — | ↑ | — | ↑ | — | ↑ | ↑ | ↑ | ↑ | ↑ | — | ↑ |
| GME6078 | putative polyketide synthase | — | — | — | — | — | — | — | ↓ | ↓ | ↓ | — | ↓ |
| GME7032 | lovastatin nonaketide synthase | ↓ | — | ↓ | — | — | — | ↓ | — | ↓ | ↑ | — | — |
| GME7327 | putative polyketide synthase | — | ↓ | — | ↓ | — | — | — | ↓ | — | ↓ | — | ↓ |
| GME7426 | hybrid PKS-NRPS | — | — | — | — | ↑ | ↑ | ↑ | — | — | — | — | — |
| GME3088 | NPRS 6 | — | — | — | — | — | — | — | — | ↑ | ↑ | — | — |
| GME5900 | NPRS 1 | — | — | — | — | — | — | — | — | — | ↑ | — | — |
| GME668 | NPRS 8 | — | — | — | ↓ | — | — | ↑ | — | — | ↓ | ↓ | — |
| GME7026 | Putative NPRS | ↓ | — | ↓ | — | — | — | ↓ | — | ↓ | ↓ | ↓ | — |
| GME7124 | Putative NPRS | — | — | — | — | — | — | — | — | ↓ | — | — | — |

1*: the gene expression difference between the mutant and M7 which fermented for 3 d; 2* : the gene expression difference between the mutant and M7 which fermented for 7 d; -: no difference; ↑: up-regulated; ↓: down-regulation.
